# Computer-Aided Decision Support Systems of Alzheimer's Disease Diagnosis - A Systematic Review

**DOI:** 10.2174/0115734056359358250516101749

**Published:** 2025-06-03

**Authors:** Tuğba Günaydın, Songül Varlı

**Affiliations:** 1 Department of Computer Engineering, Yıldız Technical University, Istanbul, Türkiye

**Keywords:** Alzheimer’s Disease, Computer Vision, Computer-Aided Diagnosis Systems, Convolutional Neural Networks, Machine Learning, Random Forest, PRISMA, STARD

## Abstract

**Background and Objective:**

The incidence of Alzheimer’s disease is rising with the increasing elderly population worldwide. While no cure exists, early diagnosis can significantly slow disease progression. Computer-aided diagnostic systems are becoming critical tools for assisting in the early detection of Alzheimer’s disease. In this systematic review, we aim to evaluate recent advancements in computer-aided decision support systems for Alzheimer’s disease diagnosis, focusing on data modalities, machine learning methods, and performance metrics.

**Methods:**

We conducted a systematic review following the Preferred Reporting Items for Systematic Reviews and Meta-Analyses guidelines. Studies published between 2021 and 2024 were retrieved from PubMed, IEEEXplore and Web of Science, using search terms related to Alzheimer’s disease classification, neuroimaging, machine learning, and diagnostic performance. A total of 39 studies met the inclusion criteria, focusing on the use of Magnetic Resonance Imaging, Positron Emission Tomography, and biomarkers for Alzheimer’s disease classification using machine learning models.

**Results:**

Multimodal approaches, combining Magnetic Resonance Imaging with Positron Emission Tomography and Cognitive assessments, outperformed single-modality studies in diagnostic accuracy reliability. Convolutional Neural Networks were the most commonly used machine learning models, followed by hybrid models and Random Forest. The highest accuracy reported for binary classification was 100%, while multi-class classification achieved up to 99.98%. Techniques like Synthetic Minority Over-sampling Technique and data augmentation were frequently employed to handle data imbalance, improving model generalizability.

**Discussion:**

Our review highlights the advantages of using multimodal data in computer-aided decision support systems for more accurate Alzheimer’s disease diagnosis. However, we also identified several limitations, including data imbalance, small sample sizes, and the lack of external validation in most studies. Future research should utilize larger, more diverse datasets, include longitudinal data, and validate models in real-world clinical trials. Additionally, explainability is needed in machine learning models to ensure they are interpretable and reliable in clinical settings.

**Conclusion:**

While computer-aided decision support systems show significant promise in improving the early diagnosis of Alzheimer’s disease, further work is needed to enhance their robustness, generalizability, and clinical applicability. By addressing these challenges, computer-aided decision support systems could play a key role in the early detection of Alzheimer’s disease and potentially reduce health care costs.

## 1 INTRODUCTION

Alzheimer’s disease (AD) is the most common form of dementia, accounting for 60-80% of dementia cases worldwide. It primarily affects memory, behavior, and cognitive abilities. Although it is not a curable disease, early diagnosis can significantly slow its progression, improving the quality of life for patients and their families. This makes early detection crucial in AD management^1^.

Patients with AD typically progress through different stages, starting from a normal cognitive state (NC) or cognitively normal (CN) to mild cognitive impairment (MCI) and, finally, Alzheimer’s disease. MCI is a critical stage, as some patients will develop AD while others remain stable. Accurately distinguishing between progressive mild cognitive impairment (pMCI) and stable mild cognitive impairment (sMCI) is especially important because early intervention at the MCI stage can delay or prevent the onset of full-blown AD [1].

Detection of the patient in the MCI stages before the diagnosis of AD is much more important than distinguishing whether the patient has AD or NC [2]. Particularly, distinguishing between p-MCI and s-MCI is critical for identifying patients at higher risk of developing AD [3].

Various diagnostic techniques are used to detect AD and its precursors [4], including:

- **Neuroimaging:** Techniques like magnetic resonance imaging (MRI) and positron emission tomography (PET) help identify brain regions affected by AD.
- **Biomarkers:** These include proteins in cerebrospinal fluid, clinical assessments, vital signs, etc., that indicate the presence of AD.
- **Genetic Risk Profilers:** These help predict AD risk by analyzing specific genetic markers.

Recent advancements in computer-aided diagnostic systems (CADs) have greatly enhanced the accuracy and speed of AD diagnosis. By combining neuroimaging data with machine learning algorithms, these systems offer a powerful tool for early detection. CAD systems can process large patient data, improving diagnostic accuracy beyond traditional methods.

Combining neuroimaging techniques with biomarkers offers a powerful and innovative approach to improving the accuracy of Alzheimer’s disease diagnosis. Biomarkers, such as those found in cerebrospinal fluid (CSF), provide protein-level insights, while neuroimaging reveals structural and functional changes in the brain [5]. By integrating these modalities, clinicians can better identify patients with mild cognitive impairment (MCI) who are at higher risk of progressing to Alzheimer’s disease. This approach enables earlier and more reliable diagnosis, setting this study apart from traditional methods that rely on a single diagnostic modality.

As of 2015, an estimated 46 million people worldwide were living with Alzheimer’s disease, a number that continues to grow annually [6]. In the United States alone, the annual cost of all types of dementia is approximately $200 billion [7a], a financial burden on par with cancer and heart disease. Beyond the economic impact, AD profoundly affects the quality of life of both patients and their families. Developing accurate and efficient diagnostic tools can help reduce the overall financial costs and improve the well-being of those affected by this debilitating disease.

According to the most recent publication from the GBD 2019 Dementia Forecasting Collaborators (2022) [7b] which draws on findings from a 2019 study and encompasses both current data and future projections about dementia-the global population of individuals with dementia, estimated at 57.4 million in 2019, is projected to rise to 152.8 million (ranging between 130.8 and 175.9 million) by 2050. This rise is primarily attributed to the global aging population and demographic changes resulting from overall population growth.

In AD diagnosis, as with many health conditions, the development of reliable and accurate systems is crucial for lowering costs and enhancing patient outcomes. This paper presents a systematic review of recent advancements in computer-aided diagnostic systems (CADs) for Alzheimer’s disease. The review focuses on the data modalities and methodologies employed in these studies, as well as the performance and generalizability of the systems reviewed.

This study is organized as follows: Section 2 describes the methodology used for selecting and analyzing the publications included in this review. Section 3 presents the results, categorizing the studies based on the data modalities, diagnostic methods, and performance metrics employed. Finally, Section 4 presents a discussion of the findings and their implications for future research and clinical application.

## 2 METHODS

This systematic review was conducted following the Preferred Reporting Items for Systematic Reviews and Meta-Analyses (PRISMA) [8] guidelines to ensure transparency and reliability in selecting and reviewing studies. Additionally, we added some elements of the Standards for Reporting Diagnostic Accuracy Studies (STARD) [9a] to assess the diagnostic accuracy of the included publications.

### 2.1 Research Questions and Aims

The objective of this review is to evaluate the performance of computer-aided diagnostic systems (CADs) for Alzheimer’s disease. We focused on answering the following key questions:

- How many studies have been published in the last three years using medical imaging data for binary or multi-class classification of Alzheimer’s disease?
- What imaging modalities (*e.g.*, MRI, PET) and machine learning algorithms were used in these studies?

- Which classification methods showed the highest accuracy, precision, recall, F1-score, and overall performance for Alzheimer’s diagnosis?
- Were there any issues related to data imbalance, and what strategies were employed to address them?
- How were the datasets divided into training and testing sets, and were there measures to prevent bias?
- Did studies report additional metrics such as AUC (Area Under the Curve) or ROC (Receiver Operating Characteristic) curves alongside accuracy?
- How were false positives and false negatives assessed in these studies?
- How generalizable were the models?

### 2.2 Study Search Methodology

A systematic search was conducted using the PubMed, IEEEXplore and Web of Science databases. All databases were queried using advanced search features, and the final search was performed on October 7, 2024. The search query combined key terms related to Alzheimer’s disease, imaging modalities, machine learning, and diagnostic performance. The full query used is as follows:

(“alzheimer’s disease” OR “ad”) AND (“multi-class classification” OR “binary classifica- tion”) AND (“mri” OR “pet” OR “neuroimaging”) AND (“machine learning” OR “deep learn- ing” OR “neural networks”) AND (“accuracy” OR “precision” OR “sensitivity” OR “specificity” OR “recall” OR “f1-score” OR “auc” OR “roc curve”) AND (“data set” OR “data imbalance” OR “bias” OR “generalization” OR “dataset”)

The search was limited to studies published in English and conducted on human subjects (or human data). We focused on studies published in the last three years (since 2021).

### 2.3 Inclusion and Exclusion Criteria

We established our inclusion and exclusion criteria to ensure the selection of high-quality, relevant studies specifically focused on Alzheimer's disease (AD) classification using neuroimaging data. Below, we provide a detailed outline of these criteria along with brief justifications. *(Inclusion-Exclusion explanation)*

#### 2.3.1 Inclusion Criteria

Studies were screened based on the following inclusion criteria:

- The publication must be a research article. Research articles are better suited to providing the essential data required for methodological comparisons and performance evaluations.
- The study must focus on the diagnosis of Alzheimer’s disease (AD), mild cognitive impairment (MCI), cognitively normal (CN), or other related dementia stages (*e.g.*, mild, moderate, non-demented). Our review specifically focuses on AD and its precursor or related stages to ensure consistency and comparability across studies.
- At least one imaging modality, such as MRI or PET, must be used for classification. Our goal is to assess computer-aided diagnostic systems that utilize neuroimaging data, a key component in AD diagnosis.
- The study must report accuracy and at least one additional metric, such as sensitivity, specificity, or AUC. Relying on a single performance metric (*e.g.*, accuracy) may be misleading to provide a comprehensive assessment of model performance, particularly in the presence of imbalanced datasets.
- The work must be published within the last three years (since 2021). We aim to capture the latest advancements in machine learning and neuroimaging techniques for AD.
- Only human data was considered (no animal studies). To ensure clinical applicability and align with the STARD guidelines, we exclude studies involving animal models, focusing only on human diagnostic accuracy assessments.

#### 2.3.2 Exclusion Criteria

Studies were screened based on the following exclusion criteria:

- Studies focusing on diseases other than AD or without neuroimaging data. The scope is limited to Alzheimer's disease diagnosis and imaging-based approaches directly related to it.
- Studies that report fewer than two performance metrics (*e.g.*, accuracy along with sensitivity, specificity, or AUC) are excluded. A comprehensive assessment of classification performance necessitates a multi-metric evaluation.
- Studies that lack sufficient methodological details, such as unclear data preprocessing or missing information on training and test splits, are excluded. Methodological transparency is essential for ensuring reproducibility and assessing the reliability of reported results.
- Review articles, conference abstracts, and other non-peer-reviewed content are excluded from consideration. Peer-reviewed research articles are better suited for providing validated scientific findings appropriate for systematic review.

The studies were screened in two phases: (1) title and abstract screening and (2) full-text screening. At the corresponding stage, those not meeting the specified criteria were excluded.

### 2.4 Data Extraction, Synthesis, and Quality Assessment

Following the selection of the final set of studies based on the aforementioned criteria, we proceeded with detailed data extraction and quality assessment.

#### 2.4.1 Quality Assessment of Included Studies

To assess the methodological quality of each included study, we evaluated the following key domains:

- Study Design: Do the studies examine whether they employed a cross-sectional or longitudinal approach, integrated control groups, and maintained transparency in participant inclusion? These topics have been discussed in the context of all studies.
- Sample Size and Diversity: The studies detailed the overall number of participants or images and described the distribution across diagnostic categories (*e.g.*, AD *vs.* CN). Demographic data were not reported individually for each study since the age ranges and gender distributions were largely the same across most of the studies.
- Machine Learning Validation/Generalization Strategy: The included studies were analyzed in terms of the validation approach used (*e.g.*, k-fold cross-validation, external validation, or train-test split), the measures implemented to prevent data leakage, and the use of hyperparameter tuning.
- Performance Reporting: It was examined whether multiple evaluation metrics (*e.g.*, accuracy, precision, recall, F1 score, AUC) were reported.
- Risk of Bias: Factors that could contribute to overfitting or biased outcomes-such as small sample sizes, single-center datasets, or insufficient explanations of data processing-were addressed across all studies.

Although we did not fully adopt a single standardized scale, we tabulated the studies by applying these criteria. The (Table **1**) included each study’s explainability, generalizability, database used, sample size used, and performance metrics. From this, conclusions were drawn and all studies were interpreted according to these criteria.

#### 2.4.2 Data Extraction and Synthesis

For each included study, we extracted the following data:

- **Study Details:** Year of publication, authors, and study design.
- **Data Modalities:** Type of imaging used (MRI, PET, etc.) or other data types such as cognitive scores.
- **Machine Learning (Classification) Methods:** Classification algorithms used (*e.g.*, CNN, Random Forest).
- **Performance Metrics:** Accuracy, sensitivity, specificity, precision, recall, F1-score, AUC, and/or ROC curves.
- **Data Handling:** How datasets were split (training/testing), and whether methods like cross-validation or oversampling were used to address data imbalance.

We synthesized the results by categorizing studies based on imaging modality and machine learning algorithms, allowing for a comparative analysis of their performance across reported metrics. Additionally, we determined whether studies included external validation sets or relied solely on internal cross-validation.

##### 2.4.2.1 Dataset Descriptions

Given the widespread use of publicly available neuroimaging databases, we documented the datasets utilized in each study (*e.g.*, ADNI (https://adni.loni.usc.edu/), Kaggle (https://www.kaggle.com/), OASIS (https://sites.wustl.edu/oas isbrains/)), along with relevant details such as sample size, patient demographics, and dataset-specific inclusion criteria.

- ADNI (Alzheimer’s Disease Neuroimaging Initiative): A widely used dataset containing longitudinal MRI and PET scans from individuals diagnosed with AD, MCI, or CN. ADNI is often leveraged for its extensive sample size (ranging from hundreds to thousands of scans) and demographic representation.
- Kaggle: Includes various smaller MRI-based AD datasets, frequently used for benchmarking purposes. Many Kaggle datasets contain only a few hundred images, often requiring augmentation or oversampling to address data limitations.
- OASIS (Open Access Series of Imaging Studies): Offers both cross-sectional and longitudinal MRI data focused on healthy aging and dementia. Sample sizes vary (typically a few hundred subjects), with demographic details such as age range and sex distribution commonly available.

#### 2.4.3 Handling of Class Imbalance

Given that class imbalance is a common challenge in Alzheimer’s disease classification (*e.g.*, fewer AD cases compared to CN, or vice versa), we systematically documented how each study handled this issue. The identified approaches included:

- Oversampling Techniques (*e.g.*, SMOTE [9b], ADASYN [9c]): Studies that generated synthetic samples for the minority class to enhance training balance.
- Data Augmentation: Particularly in MRI-based analyses, some studies applied transformations such as rotations, flips, or other geometric modifications to augment the dataset. If the method is not specifically stated, studies reported as data augmentation have performed oversampling or undersampling.
- Class Weighting: Adjusting loss functions to assign higher penalties for misclassifications in minority classes, improving model sensitivity.
- No Specific Handling (Not mentioned): Some studies did not report any method for addressing class imbalance, potentially affecting the generalizability of their findings.

Most studies reported their performance results using these methods. In some cases, since the dataset was selected as balanced, classification studies were conducted without using any additional methods. However, some studies, due to using very little data for classification, require further clarification regarding issues of bias and generalizability.

#### 2.4.4 Performance Metrics and Evaluation

To ensure a comprehensive evaluation of classification performance, we considered multiple metrics, including:

- Accuracy: The proportion of correctly classified instances. While commonly used, it can be misleading in imbalanced datasets.
- Precision, Recall, and F1-score: Particularly crucial for imbalanced class distributions. The F1-score, as the har-monic mean of precision and recall, provides a balan-ced assessment of both false positives and false negatives.
- AUC (Area Under the ROC Curve): A robust metric that evaluates model performance across various threshold settings, making it less sensitive to class imbalance.

By gathering these metrics, we aimed to compare the performance of different machine learning algorithms and data-handling strategies under varying levels of class imbalance.

## 3 RESULTS

**Fig. (1):**
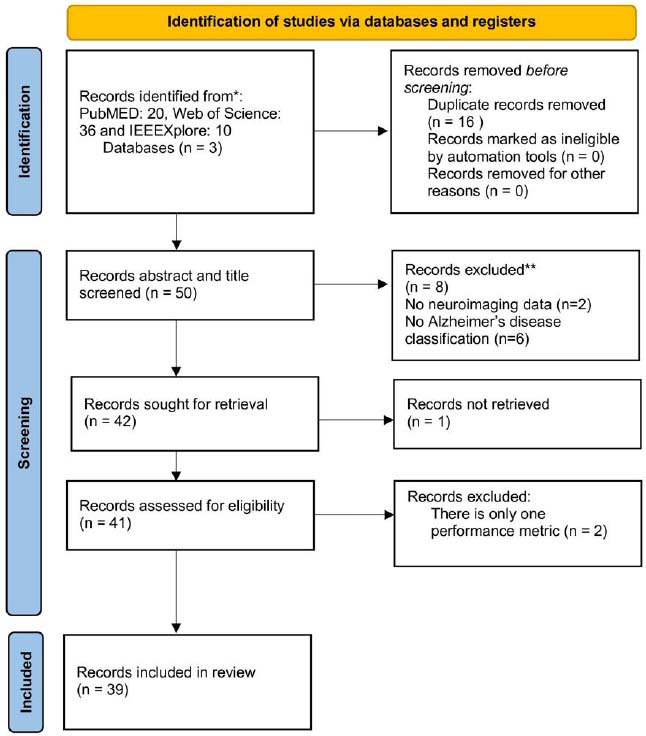
PRISMA flow diagram.

A total of 66 studies were initially identified through database searches. After applying the inclusion and exclusion criteria described in Section 2, 39 studies were selected for this systematic review. As shown in Fig. (**1**), 8 studies were excluded during the abstract and title screening, and an additional 2 studies were removed after full-text review. The PRISMA flow diagram presents this final count of 39 studies (Fig. **1**).

### 3.1 Study Design Quality and Sample Size Observations

Aligned with our Quality Assessment criteria (Section 2.4.1), we observed varying levels of methodological quality among the included studies:

#### 3.1.1 Study Design

The majority of studies employed a cross-sectional approach, while a few utilized longitudinal data, such as ADNI’s multi-timepoint scans. Studies including longitudinal data provided deeper insights into disease progression but required more complex modeling frameworks.

#### 3.1.2 Sample Size and Diversity

Sample sizes varied significantly, ranging from fewer than 100 scans (commonly in Kaggle-based datasets) to thousands of images (particularly in ADNI). While some studies justified their sample sizes using power analysis or external references, many did not explicitly assess statistical sufficiency. Studies with smaller sample sizes often relied on data augmentation or oversampling techniques to managed data limitations.

#### 3.1.3 Risk of Bias and Methodological Transparency

A subset of studies, particularly those relying on single-center data or lacking clarity in validation strategies, indicated a higher risk of bias. In contrast, studies using multi-center datasets (*e.g.*, ADNI, OASIS) and those providing transparent reporting of preprocessing steps tended to produce more robust and reproducible results.

### 3.2 Datasets

The majority of the reviewed studies utilized well-established, publicly available datasets for training and testing their models. The most frequently cited datasets were:

#### 3.2.1 Alzheimer’s Disease Neuroimaging Initiative (ADNI)^2^

Approximately 65% (only ADNI: approx 45%) of the included studies relied on ADNI, which provides large-scale MRI and PET data, along with cognitive scores and genetic information. Its longitudinal design and diverse demographic representation make it a benchmark dataset for Alzheimer’s research.

#### 3.2.2 Kaggle Datasets^3^

Around 33% of the studies used smaller, Kaggle datasets. These datasets typically contained only a few hundred MRI scans labeled by dementia stage, often requiring data augmentation or oversampling to address lack of data.

#### 3.2.3 Open Access Series of Imaging Studies (OASIS)^4^

Roughly 8% of the studies utilized OASIS, which includes MRI data from both healthy older adults and individuals with dementia. OASIS is particularly valuable for tracking cognitive decline over time in longitudinal analyses.

### 3.3 Data Modalities

The majority of studies (92,3%) relied exclusively on magnetic resonance imaging (MRI) for Alzheimer’s disease detection. However, a subset of studies (approx. 7%) employed multimodal approaches, integrating MRI with additional modality, including:

- **Positron Emission Tomography (PET):** Captures metabolic or amyloid changes associated with AD progression.
- **Cognitive test scores, genetic data, vital signs, demographics, *etc*.:** Enhance classification performance, particularly for borderline or early-stage cases.

**Fig. (2):**
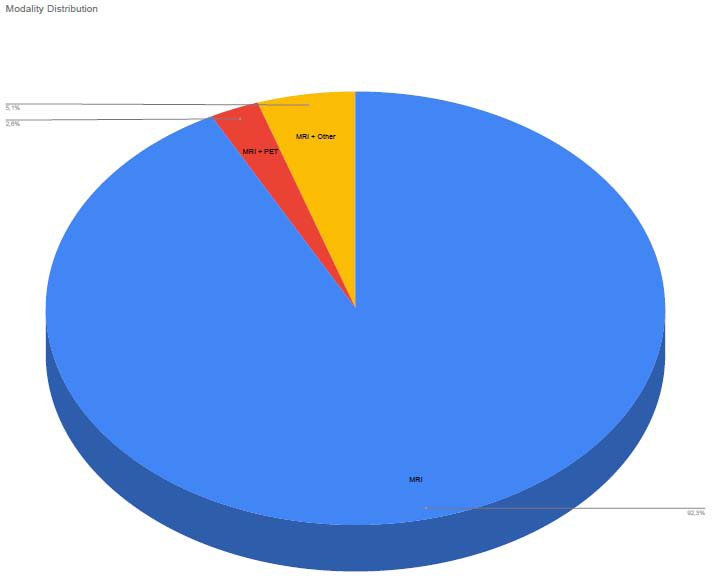
Modality distribution.

Studies including multimodal data generally reported higher diagnostic accuracy, highlighting the advantages of combining structural, functional, and other relevant information for more precise and early AD detection (Fig. **2**).

### 3.4 Classification Methods

A range of machine learning (ML) algorithms and deep learing approach (generally CNN) were employed across the 39 studies.

#### 3.4.1 Convolutional Neural Networks (CNN)

The most widely used approach for image-based classification. CNNs are highly effective at extracting features from MRI scans, allowing them to capture subtle structural changes associated with AD.

#### 3.4.2 Random Forest (RF)

Frequently applied in multi-modal studies combining imaging with clinical data. RF is valued for its robustness to overfitting and its ability to provide interpretable feature importance insights.

#### 3.4.3 Support Vector Machine (SVM)

Particularly suited for high-dimensional neuroimaging data. SVMs demonstrated strong performance in smaller datasets, provided that appropriate feature selection or dimensionality reduction techniques were implemented.

#### 3.4.4 Hybrid Models

These approaches combined CNNs with other algorithms (*e.g.*, SVM, RF) to exploit complementary strengths. Hybrid models often reported performance improvements, particularly in multi-class classification tasks.

Each model’s performance depended on the type of data and the classification task (binary *vs.* multi-class classification). The distribution of the methods used by studies is in Fig. (**3**).

2
https://adni.loni.usc.edu/
3
https://www.kaggle.com/
4
https://sites.wustl.edu/oasisbrains/


#### 3.4.5 Algorithmic Performance and Limitations

CNNs generally achieved the highest accuracy, especially in binary classifications (*e.g.*, AD *vs.* CN). However, their performance declined when training data were insufficient or highly imbalanced. Random Forest demonstrated stable performance when integrating imaging with clinical or demographic variables, but it required careful hyperparameter tuning. SVMs were effective for smaller datasets but were highly sensitive to parameter selection (*e.g.*, kernel type) and data preprocessing strategies. Hybrid Models offered improved performance but introduced greater complexity and longer training times, requiring careful implementation for optimal results.

### 3.5 Performance Metrics

**Fig. (4):**
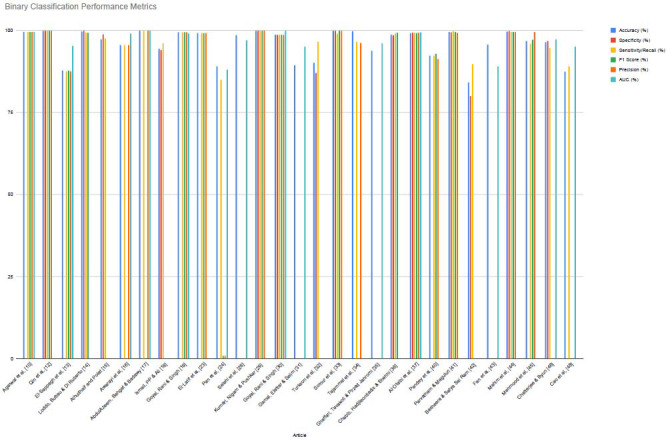
Binary classification performance metrics by the studies.

**Fig. (5):**
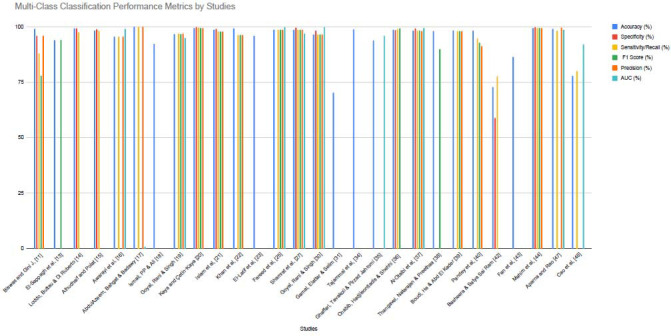
Multi-class classification performance metrics by the studies.

While accuracy was the most commonly reported performance metric, many studies also included precision, recall, F1-score, and AUC to provide a more comprehensive evaluation. We analyzed binary and multi-class classification performance separately (Figs. **4** and **5**).

Best performances according to accuracy:

- **Best Binary Classification:** Qin *et al*. [12] achieved 100% accuracy for distinguishing aMCI from sMCI using a 3D CNN with hybrid attention (3D HA-ResUNet) applied to MRI data.
- **Best Multi-class Classification:** AbdulAzeem, Bahgat, and Badawy [17] reported 99.98% accuracy for AD *vs.* NC *vs.* MCI using a CNN-based model trained on MRI scans.

Although reported accuracy values were often high, studies that included additional metrics such as F1-score, precision, recall, and AUC provided stronger evidence of model robustness, particularly in the presence of class imbalance. Notably, multimodal studies demonstrated improved performance, likely due to the complementary nature of imaging and non-imaging features.

### 3.6 Handling of Data Imbalanced

Data imbalance, such as a disproportionately higher number of CN cases compared to AD, posed a significant challenge in many studies. To handle this issue and to increase the generalization ability of the model, various strategies were employed:

- Oversampling (*e.g.*, SMOTE, ADASYN):

Synthetic samples were generated to balance class distributions. While effective in certain cases, this method risked amplifying noise, especially in small or highly heterogeneous datasets.

- **Data Augmentation:**

Image transformations such as rotation, flipping, and scaling were applied to expand training datasets and reduce overfitting, particularly in CNN-based models.

- **Cross Validation:** Cross-validation splits the dataset into multiple subsets, allowing the model to be evaluated on each segment, which helps reduce the risk of overfitting. Techniques like stratified k-fold cross-validation maintain the representation of the minority class by preserving its distribution in every fold, especially in imbalanced datasets. This approach enables a more objective and reliable assessment of the model's overall performance.

- **Class Weighting:**

A subset of studies adjusted loss functions to assign higher penalties for misclassifications in underrepresented classes. This approach provided a computationally efficient alternative to oversampling.

- **No Specific Handling:**

Some studies did not report any strategy for addressing class imbalance, potentially limiting their models’ generalizability and performance in real-world clinical applications.

Because methods ranged from simple augmentation to more advanced GAN-based approaches, comparing effectiveness was challenging. Nonetheless, studies that explicitly addressed imbalance generally reported more stable metrics (*e.g.*, higher F1-scores and fewer false negatives), underscoring the importance of proper class balancing techniques.

These methods played a crucial role in ensuring that the models could generalize across different patient populations and perform well even with imbalanced datasets.

**Fig. (6):**
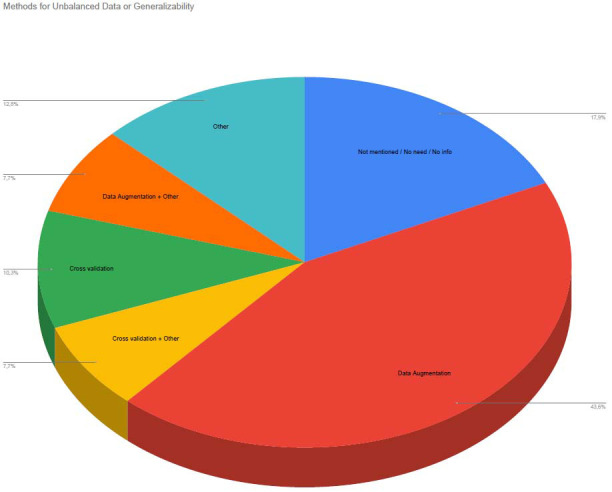
Used methods for unbalanced data (and also generalizability).

In some of the studies reviewed, precautions were taken for unbalanced class distributions (Fig. **6**).

Most reviewed studies demonstrated a high ability to diagnose Alzheimer’s disease using structural MRI images. Typically, the binary classification of AD versus CN (healthy controls) achieved accuracy levels between 90% and 100%, with some deep learning models performing nearly perfectly. In contrast, determining the MCI (Mild Cognitive Impairment) stage-and particularly distinguishing between its subtypes (sMCI *vs.* pMCI)-proved more challenging, with accuracies generally between 75% and 90% (*e.g.*, sensitivities of 80–95% for distinguishing AD from MCI). For multi-class problems (such as differentiating among AD, MCI, and CN or additional stages), deep learning models typically reached accuracies between 85% and 97%, and several studies even reported overall accuracies exceeding 90% for three- or four-class classification tasks. Overall, deep learning-based approaches have outperformed classical machine learning methods (*e.g.*, SVM, Random Forest), though traditional methods have also been successfully applied on smaller datasets-achieving accuracies of up to 90% when combined with effective feature extraction techniques.

## 4 DISCUSSION

This review highlights a diverse range of computer-aided diagnostic (CAD) approaches for Alzheimer’s disease (AD) classification, showcasing their potential to enhance early detection. While these findings emphasize the promise of CAD systems, they also expose critical methodological and practical challenges that must be addressed to achieve reliable and clinically meaningful outcomes.

### 4.1 Methodological Quality and External Validation *(Critique of Methodological Quality, External Validation, Model Explainability, Data Imbalance Strategies, and Limitations*.

A key finding of this review was the variability in methodological quality across the 39 included studies. While many utilized well-established datasets such as ADNI and OASIS, the majority relied on cross-sectional designs. Only a small subset used longitudinal analyses, which are essential for tracking disease progression from mild cognitive impairment (MCI) to Alzheimer’s disease (AD).

#### 4.1.1 External Validation Gaps

Most studies depended only on internal validation methods, such as k-fold cross-validation, without evaluating their models on external datasets. This lack of external validation raises concerns regarding model generalizability and robustness in real-world clinical settings. To ensure that reported accuracies are not due to overfitting or dataset-specific artifacts, future research should prioritize:

- Independent test sets
- Multi-center datasets
- External validation cohorts

#### 4.1.2 Sample Size Justification

Although some studies used power analyses or referenced external benchmarks to justify their sample sizes, many did not provide explicit rationale. Small or highly homogeneous samples can significantly limit the generalizability of findings. To enhance the clinical applicability of CAD systems, future studies should include:

- Larger and more diverse datasets.
- Representation of different disease stages.
- Strategies to reduce bias and ensure robust model performance across varied clinical populations.

### 4.2 Data Imbalance Strategies and Performance Assessments

Data imbalance, such as a disproportionately higher number of cognitively normal (CN) compared to Alzheimer’s disease (AD) cases, was a recurring challenge across the reviewed studies. Various techniques were employed to address this issue, including SMOTE, ADASYN, cross-validation, and data augmentation to artificially expand the minority class, while class weighting provided a simpler alternative to account for imbalanced distributions.

#### 4.2.1 Effectiveness *vs.* Overfitting

While these techniques often led to improvements in reported performance metrics-such as higher F1-scores and reduced false negatives-they also had potential risks. If not carefully applied, oversampling and augmentation methods can amplify noise or generate synthetic artifacts, particularly in small or heterogeneous datasets. To ensure robustness, future studies should:

- Evaluate the long-term stability of imbalance-handling methods
- Include external validation to assess generalizability
- Use additional metrics (*e.g.*, confusion matrices) to detect potential overfitting

#### 4.2.2 GAN-Based Augmentation

A small subset of studies (only Goyal, Ran & Singh (2024)) explored Generative Adversarial Networks (GANs) for data augmentation, allowing for the generation of more realistic synthetic samples. However, GAN-based methods also pose risks, particularly when trained on small datasets, as they may produce unrealistic or redundant images. This highlights the importance of:

- Transparent reporting of GAN-generated data
- Thorough validation to ensure the synthetic samples meaningfully enhance model performance rather than introducing bias.

### 4.3 Model Explainability and Clinical Applicability

As computer-aided diagnostic (CAD) systems become increasingly complex, particularly with the adoption of deep neural networks, explainability is crucial for clinical acceptance. While some studies (11 of 39) explored interpretability tools (*e.g.*, Grad-CAM, SHAP), most did not provide detailed explanations of how predictions were generated.

#### 4.3.1 Importance of Interpretability

Clinicians require a clear rationale behind each classification to trust automated decisions, especially for high-stakes diagnoses such as Alzheimer's disease. Future models should include interpretability techniques such as:

- Grad-CAM (Gradient-weighted Class Activation Mapping)
- Layer-wise Relevance Propagation (LRP)
- Shapley Values (SHAP)

These methods can help identify which regions of brain scans contribute most to a positive AD classification, improving transparency and trust in AI-driven diagnostic tools.

#### 4.3.2 Integration with Clinical Workflows

Beyond technical performance, CAD systems must smoothly integrate into existing diagnostic pipelines. Standardized data acquisition protocols, preprocessing workflows, and user-friendly interfaces can facilitate adoption and enhance clinical utility.

### 4.4 Overfitting, Data Leakage, and Extremely High Accuracy Reports

Several studies reported near-perfect classification accuracy (*e.g.*, 99–100%), particularly in binary AD *vs.* CN tasks. While such results may seem promising, they often raise concerns about potential methodological flaws, including:

- Overfitting: Models trained on small or imbalanced datasets may memorize patterns rather than learning generalizable features.
- Data Leakage: Unintentional inclusion of test data in the training process (*e.g.*, overlapping patient IDs in train/test splits) can artificially inflate performance.
- Lack of External Validation: Without independent test sets, it remains unclear whether these models generalize to diverse patient populations.

#### 4.4.1 Clinical Feasibility Check

Studies reported 100% accuracy often lacked additional validation through external cohorts or prospective clinical trials, requiring careful interpretation of results. Future research should:

- Cross-validate models on independent multi-center datasets.
- Systematically check for potential data leakage in data partitioning strategies.
- Ensure that reported accuracies demonstrate real-world diagnostic utility rather than dataset-specific artifacts.

### 4.5 Future Research Directions

Building upon the limitations observed in this review, we propose several strategies to advance CAD systems for AD classification:

#### 4.5.1 Robust Data Collection and Sharing

- Encourage multi-institution collaborations to assemble larger, more diverse datasets.
- Expand public database like ADNI, OASIS, and Kaggle to include more comprehensive demographic and longitudinal data coverage.

#### 4.5.2 Longitudinal Modeling

- Focus on tracking disease progression (*e.g.*, MCI to AD) using time-series or sequence-based models.
- Identify early biomarkers that predict AD conversion through longitudinal analysis.

#### 4.5.3 Explainable AI Frameworks

- Integrate model-agnostic interpretability tools (*e.g.*, LIME, SHAP) and deep-learning-specific methods (*e.g.*, Grad-CAM) to improve clinical trust.
- Facilitate regulatory approval by ensuring AI decisions are explainable and reproducible.

#### 4.5.4 Standardization of Protocols

- Establish common preprocessing pipelines (*e.g.*, skull stripping, intensity normalization, registration) to enhance reproducibility.
- Encourage consistent performance reporting (*e.g.*, confusion matrices, calibration plots) to enable direct comparisons across studies.

#### 4.5.5 Prospective Clinical Trials

- Conduct real-world evaluations of high-performing CAD systems to assess clinical impact, including:
  - Time to diagnosis
  - Cost-effectiveness
  - User acceptability in healthcare settings

### 4.6 Limitations of This Study

Despite synthesizing a wide range of studies, this review has several limitations:

#### 4.6.1 Publication Bias

- Only peer-reviewed articles in English from the last three years were included, which may have excluded relevant but unpublished or non-English research.

#### 4.6.2 Search Scope

- While multiple databases were searched (PubMed, IEEE Xplore, Web of Science), additional sources or grey literature may contain findings not captured in this review.

#### 4.6.3 Heterogeneity of Studies

- Variability in data preprocessing, model architectures, and reported metrics complicated direct comparisons.
- Although studies were grouped by modality and ML algorithm, use of a wide variety of methods in studies prevented a more detailed meta-analysis.

#### 4.6.4 Evolving Field

- Given rapid advancements in machine learning, novel techniques may have emerged since the final search date.
- Ongoing updates to public datasets and the introduction of new augmentation strategies may shift performance trends over time.

## CONCLUSION

This systematic review highlights the significant potential of computer-aided diagnostic (CAD) systems in the early detection and classification of Alzheimer’s disease (AD). By leveraging advanced machine learning algorithms applied to neuroimaging data-primarily MRI, but also multimodal inputs such as PET scans and cognitive scores-these systems achieve high diagnostic accuracy and hold promise for enhancing clinical decision-making. Notably, multimodal approaches consistently outperform single-modality models, reinforcing the importance of integrating structural, functional, and other relevant information for a more comprehensive assessment of disease progression.

## Figures and Tables

**Fig. (3):**
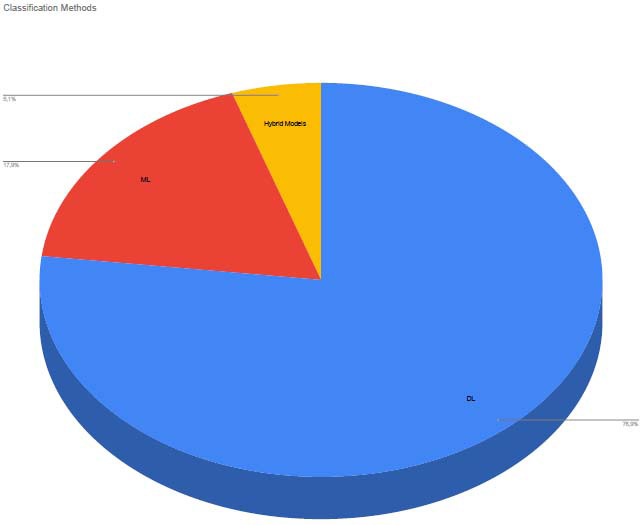
Classification methods by studies.

**Table 1:** A summary of articles included in the study.

**Article**	**Dataset**	**Imbalanced** **Data Solution/G eneralizability**	**Divided Meth-** **ods**	**Modality**	**Classification** **Type**	**Classes**	**Classification** **Methods**	**Accuracy**	**Specificity**	**Sensitivity/Recall**	**F1 Score**	**Precision**	**Area Under the** **Curve (AUC)**	**Explainable Artificial Intelligence (XAI)**
Agarwal *et al*. [10]	ADNI and IXI (Information eXtraction from Images) AD (n = 245), CN (n = 245), and sMCI (n = 229)	No need data is balanced	Stratified 5-fold cross-validation	T1 MRI	Binary Classification	AD *vs.* CN; sMCI *vs.* AD	DenseNet264 (Best Model for AD *vs.* CN), EfficientNet (B0, B1, B2, B3), DenseNet201 (Best Model for AD *vs.* sMCI), DenseNet (121,169)	AD *vs.* CN: 99.55%; sMCI *vs.* AD: 82.06%	---	AD *vs.* CN: 99.55%; sMCI *vs.* AD: 82.06%	AD *vs.* CN: 99.55%; sMCI *vs.* AD: 81.84%	AD *vs.* CN: 99.56%; sMCI *vs.* AD: 83.70%	AD *vs.* CN: 99.55%; sMCI *vs.* AD: 82.06%	Not mentioned
Biswas and Gini J [11].	ADNI and OASIS (ADNI: 899, OASIS: 322)	Not mentioned (no info about distribution of classes)	75% training, 25% testing	3D MRI	Multi-class Classification	Normal *vs.* Mild AD *vs.* Severe AD	Random Forest (Best Model for OASIS), Gradient Boost (Best Model for ADNI), Decision Tree, KNN	99% (RF), 92% (GB)	96% (RF), 52%(GB)	88% (RF), 83%(GB)	78% (RF), 65% (GB)	96% (RF), 83% (GB)	-	Not mentioned
Qin *et al*. [12]	ADNI (AD: 98, CN: 114) and Local Dataset (aMCI: 43, sMCI: 46, oMCI: 5)	Adjusting class-specific regularization parameters	80% Training (10% Validation) and 20% Test	T1 sMRI	Binary Classification	AD *vs.* CN; aMCI *vs.* sMCI	3D HA-ResUNet	AD *vs.* CN: 92.68%; aMCI *vs.* sMCI: 100%	AD *vs.* CN: 95.45%; aMCI *vs.* sMCI: 100%	AD *vs.* CN: 89.47%; aMCI *vs.* sMCI: 100%	AD *vs.* CN: 91.89%; aMCI *vs.* sMCI: 100%	AD *vs.* CN: 94.44%; aMCI *vs.* sMCI: 100%	-	Gradient-weighted Class Activation Mapping (Grad-CAM)
El-Sappagh *et al*. [13].	ADNI (CN: 294, sMCI: 254, pMCI: 232, AD: 268)	SMOTE	10-fold-cross validation	Cognitive scores, MRI, PET, Genetics, Medical history (Lab tests, demographics)	Binary and Multi-class Classification	sMCI *vs.* pMCI; CN *vs.* MCI *vs.* AD	Random Forest (RF)	sMCI *vs.* pMCI: 87.76%; CN *vs.* MCI *vs.* AD: 93.95%	---	sMCI *vs.* pMCI: 87.50%	sMCI *vs.* pMCI: 87.75%; CN *vs.* MCI *vs.* AD: 93.94%	sMCI *vs.* pMCI: 87.50%	sMCI *vs.* pMCI: 0.953	SHapley Additive exPlanations (SHAP)
Loddo, Buttau, and Di Ru- berto [14]	ADNI MRI (NC: 213, sMCI: 90, pMCI: 126, AD: 130), ADNI-2 fMRI (NC: 433, EMCI: 431, LMCI: 354, MCI: 50, SMC: 68, AD: 198) OASIS (CN: 1742, mild dementia: 137, very mild dementia: 340, moderate dementia: 10), Kaggle (2560 healthy subjects, very mild dementia: 1792, mild dementia: 717, moderate dementia: 52)	Data augmentation	80% training, 10% validation, and 10% testing for MRI; 30% training, 20% validation, and 50% testing for fMRI	MRI and fMRI	Binary and Multi-class Classification	AD *vs.* Normal Control (NC); NC *vs.* Very Mild Dementia *vs.* Mild Dementia *vs.* Moderate Dementia (OASIS, Kaggle); NC *vs.* MCI *vs.* AD (ADNI)	Deep-ensemble method combining features from CNN architectures (AlexNet, ResNet-50, ResNet-101, GoogLeNet, Inception-ResNet-v2)	NC *vs.* AD: 98.51% (OASIS), 96.57% (Kaggle), 99.74% (ADNI); NC *vs.* MCI *vs.* AD: 99.22% (ADNI); NC *vs.* very mild AD *vs.* mild AD *vs.* moderate AD: 98.24% (OASIS), 97.71% (Kaggle)	NC *vs.* AD: 98.42% (OASIS), 99.28% (Kaggle), 99.89% (ADNI); NC *vs.* MCI *vs.* AD: 99.20% (ADNI); NC *vs.* very mild AD *vs.* mild AD *vs.* moderate AD: 97.31% (OASIS), 98.22% (Kaggle)	NC *vs.* AD: 97.57% (OASIS), 96.57% (Kaggle), 99.36% (ADNI); NC *vs.* MCI *vs.* AD: 97.53% (ADNI); NC *vs.* very mild AD *vs.* mild AD *vs.* moderate AD: 93.05% (OASIS), 96.67% (Kaggle)	NC *vs.* AD: 97.85% (OASIS), 96.57% (Kaggle), 99.35% (ADNI)	-	-	Not mentioned
Alhudhaif and Po- lat [15]	Kaggle (CN: 3200, Very Mild Dementia: 2240, Mild Dementia: 1039, Moderate Dementia: 64)	Data augmentation and Fusion loss function combining Generalized Dice Loss (GDL) and Focal Loss (FL)	80% training, 20% testing and 5-fold cross-validation	T1 MRI	Binary and Multi- class Classification	No Dementia *vs.* Demented (AD); No Dementia *vs.* Very Mild Dementia *vs.* Mild Dementia *vs.* Moderate Dementia	Residual Block Fully Connected Deep Convolu- tional Neural Network (DCNN)	No Dementia *vs.* Demented (AD): 97.3%; No Dementia *vs.* Very Mild Dementia *vs.* Mild Dementia *vs.* Mod- erate Dementia: 98.2%	No Dementia *vs.* Demented (AD): 98.8%; No Dementia *vs.* Very Mild Dementia *vs.* Mild De- mentia *vs.* Moderate Dementia: 98.9%	No Dementia *vs.* Demented (AD): 97.5%; No Dementia *vs.* Very Mild Dementia *vs.* Mild Dementia *vs.* Moderate Dementia: 98.2%	-	-	-	Not mentioned
Awarayi *et al*. [16]	ADNI (AD: 1581, MCI: 1310, CN: 1591)	Data Augmentation	10-fold-cross validation	MRI	Binary and Multi-class Classification	AD *vs.* MCI *vs.* NC; AD *vs.* NC; AD *vs.* MCI; MCI *vs.* NC	Custom CNN architecture with four convolutional layers and two hidden layers	AD *vs.* MCI *vs.* NC: 93.45%; AD *vs.* MCI: 94.92%; AD *vs.* NC: 94.39%; MCI *vs.* NC: 95.62%	---	AD *vs.* MCI *vs.* NC: 93.24%; AD *vs.* MCI: 94.92%; AD *vs.* NC: 94.39%; MCI *vs.* NC: 95.62%	-	AD *vs.* MCI *vs.* NC: 93.70%; AD *vs.* MCI: 94.92%; AD *vs.* NC: 94.39%; MCI *vs.* NC: 95.62%	AD *vs.* MCI *vs.* NC: 0.99; AD *vs.* MCI: 0.98; AD *vs.* NC: 0.99; MCI *vs.* NC: 0.99	Not mentioned
AbdulAzeem, Bahgat and Badawy [17]	ADNI (No detail)	Data Augmentation	95% Training (90% Training 10% Validation), 5% Testing	MRI	Binary and Multi- class Classification	AD *vs.* CN; AD *vs.* MCI *vs.* CN	CNN-based architecture with three convolutional layers	AD *vs.* CN: 100%; AD *vs.* MCI *vs.* CN: 99.98%	-	AD *vs.* CN: 100%; AD *vs.* MCI *vs.* CN: 99.98%	-	AD *vs.* CN: 100%; AD *vs.* MCI *vs.* CN: 99.98%	AD *vs.* CN: 1.00	Not mentioned
Ismail, PP and Ali [18]	ADNI (AD: 511, MCI: 571, CN:535)	Data Augmentation, Multi-Objective Grasshopper Optimization Algorithm (MOGOA)	70% Training and 30% Testing and 10-fold cross-validation	MRI and PET	Binary and Multi- class Classification	AD *vs.* NC; MCI *vs.* NC; AD *vs.* MCI; AD *vs.* MCI *vs.* NC.	Ensemble deep learning framework (MultiAz- Net) with AlexNet, InceptionV3, and ResNet-18 via SVM	AD *vs.* NC: 94.4%; MCI *vs.* NC: 93.2%; AD *vs.* MCI: 90.00%; AD *vs.* MCI *vs.* NC: 92.3%	AD *vs.* NC: 94.0%; MCI *vs.* NC: 89.2%; AD *vs.* MCI: 93.3%	AD *vs.* NC: 95.0%; MCI *vs.* NC: 96.00%; AD *vs.* MCI: 89.2%	-	-	-	Not mentioned
Goyal, Rani and Singh [19]	ADNI (AD: 1980, MCI: 2010, CN: 1990)	Generative Adversarial Networks (GANs)	70% training, 10% validation, and 20% testing	2D MRI	Binary and Multi- class Classification	AD *vs.* CN; AD *vs.* MCI; CN *vs.* MCI; AD *vs.* MCI *vs.* CN	Transfer learning from AlexNet and a combination of Long Short-Term Memory (LSTM) networks	AD *vs.* CN: 98.13%; AD *vs.* MCI: 99.38%; CN *vs.* MCI: 99.37%; AD *vs.* MCI *vs.* CN: 96.83%	-	AD *vs.* CN: 98.13%; AD *vs.* MCI: 99.38%; CN *vs.* MCI: 99.37%; AD *vs.* MCI *vs.* CN: 96.85%	AD *vs.* CN: 98.13%; AD *vs.* MCI: 99.38%; CN *vs.* MCI: 99.37%; AD *vs.* MCI *vs.* CN: 96.83%	AD *vs.* CN: 98.15%; AD *vs.* MCI: 99.39%; CN *vs.* MCI: 99.37%; AD *vs.* MCI *vs.* CN: 96.87%	AD *vs.* CN: 0.98; AD *vs.* MCI: 0.99; CN *vs.* MCI: 0.99; AD *vs.* MCI *vs.* CN: >0.95	Not mentioned
Kaya and Çetin- Kaya [20]	Kaggle (Mild Demented: 869, Moderate Demented: 64, Non-Demented: 3200, Very Mild Demented: 2240) and ADNI	Class weighting	80% training (10% validation), 20% testing	MRI	Multi-class Classification	Mild De- mented *vs.* Moderate Demented *vs.* Non- Demented *vs.* Very Mild Demented; AD *vs.* CN *vs.* CI	Convolutional Neural Networks (CNN) with architecture optimized using Particle Swarm Optimization (PSO)	Mild Demented *vs.* Moderate Demented *vs.* Non-Demented *vs.* Very Mild Demented: 99.53%; AD *vs.* CN *vs.* CI: 99.32%	Mild Demented *vs.* Moderate Demented *vs.* Non-Demented *vs.* Very Mild Demented: 99.83%; AD *vs.* CN *vs.* CI: 99.71%	Mild Demented *vs.* Moderate Demented *vs.* Non-Demented *vs.* Very Mild Demented: 99.70%; AD *vs.* CN *vs.* CI: 99.32%	Mild Demented *vs.* Moderate Demented *vs.* Non-Demented *vs.* Very Mild Demented: 99.54%; AD *vs.* CN *vs.* CI: 99.24%	Mild Demented *vs.* Moderate Demented *vs.* Non-Demented *vs.* Very Mild Demented: 99.38%; AD *vs.* CN *vs.* CI: 98.99%	-	Not mentioned
Islam *et al*. [21]	ADNI (CN: 470, MCI: 477, AD: 599)	Duplication MRIs	80% training (10% validation), 20% testing	sMRI	Multi-class Classification	AD *vs.* MCI *vs.* CN	Support Vector Ma chine (SVM)	98.71%	99.04%	97.89%	97.92%	97.96%	-	Not mentioned
Khan *et al*. [22]	ADNI (NC: 80, EMCI: 75, LMCI: 70, AD: 75)	Data Augmentation	70% training, 20% testing, and 10% validation	MRI	Multi-class Classification	NC *vs.* EMCI *vs.* LMCI *vs.* AD	PMCAD-Net (CNN-based architecture)	99.2%	---	96.3%	96.34%	96.4%	-	Not mentioned
El-Latif *et al*. [23]	Kaggle (Mild Demented: 896, Moderate Demented: 64, Non-Demented: 3200, Very Mild Demented: 2240)	Data Augmentation	70% training, 20% testing, and 10% validation	MRI	Binary and Multi- class Classification	AD *vs.* Non- AD; Non- Demented *vs.* Very Mild Demented *vs.* Mild Demented *vs.* Moderate Demented	Lightweight CNN model with seven layers	AD *vs.* Non-AD: 99.22%; Non- Demented *vs.* Very Mild Demented *vs.* Mild Demented *vs.* Moderate Demented: 95.93%	-	AD *vs.* Non- AD: 99.22%	AD *vs.* Non- AD: 99.21%	AD *vs.* Non- AD: 99.22%	-	Not mentioned
Pan *et al*. [24]	ADNI (AD: 237, MCIc: 115, MCInc: 173, NC: 262) and OASIS (AD: 105, NC: 91)	Stratified five-fold cross validation	80% training, 20% testing	MRI	Binary Classification	AD *vs.* NC; MCIc *vs.* NC; MCIc *vs.* MCInc	3D CNN, Ensemble Learning, and Genetic Algorithm	AD *vs.* NC: 89%; MCIc *vs.* NC: 88%; MCIc *vs.* MCInc: 71%	-	AD *vs.* NC: 85%; MCIc *vs.* NC: 84%; MCIc *vs.* MCInc: 65%	AD *vs.* NC: 0.88; MCIc *vs.* NC: 0.87; MCIc *vs.* MCInc: 0.69	AD *vs.* NC: 0.90; MCIc *vs.* NC: 0.81; MCIc *vs.* MCInc: 61%	AD *vs.* NC: 88%; MCIc *vs.* NC: 87%; MCIc *vs.* MCInc: 70%	Gradient-weighted Class Activation Mapping (Grad-CAM)
Fareed *et al*. [25]	Kaggle (Non-Demented: 3200, Very Mild Demented: 2240, Mild Demented: 896, Moderate Demented: 64)	SMOTETOMEK	60% training, 20% validation, and 20% testing	MRI	Multi-class Classification	Non- Demented *vs.* Very Mild Demented *vs.* Mild Demented *vs.* Moderate Demented	ADD-Net CNN	98.63%	-	98.58%	98.61%	98.63%	99.76%	Grad-CAM
Khatri and Kwon [26]	ADNI ([Subjects] AD: 63, MCIs: 37 stabil MCI, MCIc: 45 MCI, HC: 68; subject-based)	Ten-fold cross validation	70% training and 30% testing	sMRI and rsMRI	Binary Classification	AD *vs.* HC; MCIc *vs.* HC; MCIc *vs.* MCIs; AD *vs.* MCIc *vs.* MCIs *vs.* HC	SVM (Best Model) and RF	AD *vs.* HC: 95.87%; AD *vs.* MCI: 92.45%; HC *vs.* MCI: 90.35%; MCIs *vs.* MCIc: 88.03%	AD *vs.* HC: 95.95%; AD *vs.* MCI: 91.71%; HC *vs.* MCI: 92.11%; MCIs *vs.* MCIc: 89.71%	AD *vs.* HC: 97.35%; AD *vs.* MCI: 95.98%; HC *vs.* MCI: 94.34%; MCIs *vs.* MCIc: 94.85%	AD *vs.* HC: 96.33%; AD *vs.* MCI: 93.75%; HC *vs.* MCI: 94.13%; MCIs *vs.* MCIc: 93.17%	-	AD *vs.* HC: 97.03%; AD *vs.* MCI: 94.03%; HC *vs.* MCI: 92.06%; MCIs *vs.* MCIc: 91.08%	Not mentioned
Shamrat *et al*. [27]	ADNI (after data augmentation each class has 10000 MRIs. Classes are CN, EMCI, MCI, LMCI, Subjective Memory Concern (SMC), and AD)	Data augmentation	60% training, 20% validation, and 20% testing	T2-w MRI	Multi-class Classification	NC *vs.* SMC *vs.* MCI *vs.* EMCI *vs.* LMCI *vs.* AD	AlzheimerNet, a fine-tuned InceptionV3	98.68%	99.74%	98.68%	98.68%	98.68%	0.97 (average for each class)	Grad-CAM
Salehi *et al*. [28]	Kaggle (Non-Demented: 3200, Very-Mild-Demented (AD): 2240)	Shuffle-Split Cross Validation	80% training and 20% testing	MRI	Binary Classification	Non- Demented (ND) *vs.* Very-Mild- Demented (VMD)	LSTM (Long Short- Term Memory) networks	98.62%	-	-	-	-	0.97	Not mentioned
Kumari, Nigam and Pushkar [29]	ADNI (NC: 922 MRI, 106 FDG-PET, 49 PiB-PET; MCI: 2795 MRI, 384 FDG-PET, 142 PiB-PET; AD: 465 MRI, 59 FDG-PET, 32 PiB-PET)	Stratified Shuffle-Split Cross-Validation	70% training and 30% testing	MRI, FDG-PET, PiB-PET, and cognitive assessments	Binary Classification	AD *vs.* NC; MCI *vs.* NC; AD *vs.* MCI	Adaptive Hyperparameter Tuning Random Forest En- semble (HPT-RFE)	AD *vs.* NC: 100%; MCI *vs.* NC: 91%; AD *vs.* MCI: 95%	AD *vs.* NC: 100%; MCI *vs.* NC: 100%; AD *vs.* MCI: 100%	AD *vs.* NC: 100%; MCI *vs.* NC: 60%; AD *vs.* MCI: 80%	AD *vs.* NC: 100%; MCI *vs.* NC: 75%; AD *vs.* MCI: 88.88%	AD *vs.* NC: 100%; MCI *vs.* NC: 100%; AD *vs.* MCI: 100%	-	Not mentioned
Goyal, Rani and Singh [30]	ADNI (CN: 1485, MCI: 1510, AD: 1490)	Resampling techniques (under and oversampling)	70% training (10% validation) and 30% testing	2D T1-w MRI	Binary and Multiclass Classification	AD *vs.* CN; AD *vs.* MCI; CN *vs.* MCI; AD *vs.* CN *vs.* MCI	Ensemble learning (and using ranking-based ensembled multiclassifier) with: VGG16, VGG19, ResNet50 V2, ResNet101 V2, and MobileNet	AD *vs.* CN *vs.* MCI: 96.6% (VGG16); AD *vs.* CN: 97.77% (MobileNet); AD *vs.* MCI: 96.89% (VGG19); CN *vs.* MCI: 98.66% (VGG16)	AD *vs.* CN *vs.* MCI: 98.29% (VGG16); AD *vs.* CN: 96.54% (VGG16-VGG19); AD *vs.* MCI: 96.89% (VGG19); CN *vs.* MCI: 98.68% (VGG16)	AD *vs.* CN *vs.* MCI: 96.6% (VGG16); AD *vs.* CN: 97.77% (MobileNet); AD *vs.* MCI: 96.89% (VGG19); CN *vs.* MCI: 98.65% (VGG16)	AD *vs.* CN *vs.* MCI: 96.6% (VGG16); AD *vs.* CN: 97.58% (VGG19); AD *vs.* MCI: 96.89% (VGG19); CN *vs.* MCI: 98.67% (VGG16)	AD *vs.* CN *vs.* MCI: 96.6% (VGG16); AD *vs.* CN: 97.61% (VGG19); AD *vs.* MCI: 96.89% (VGG19); CN *vs.* MCI: 98.68% (VGG16)	AD *vs.* CN *vs.* MCI: 99.82% (VGG19); AD *vs.* CN: 99.83% (MobileNet); AD *vs.* MCI: 99.75% (VGG19); CN *vs.* MCI: 99.9% (VGG16)	Not mentioned
Gamal, Elattar and Selim [31]	ADNI (789 MRI)	Data augmentation, 5-fold cross-validation	70% training (each fold has 20% validation), 30% testing	3D T1-w MRI	Binary and Multi-class Classification	AD *vs.* CN; AD *vs.* MCI; MCI *vs.* CN; AD *vs.* MCI *vs.* CN	Ensemble of 3D deep learning architectures: 3D CNN, DenseNet201, and Vision Transformer (ViT)	AD *vs.* CN: 89.46%; AD *vs.* MCI: 78.60%; MCI *vs.* CN: 78.86%; AD *vs.* MCI *vs.* CN: 70.33%	-	-	-	-	AD *vs.* CN: 95.09%; AD *vs.* MCI: 85.81%; MCI *vs.* CN: 85.63%	Not mentioned
Turkson *et al*. [32]	ADNI (AD: 150, MCI: 150, CN: 150)	No need data is balanced	86.6% training and 13.4% testing (5 fold cross-validation with 20% validation each fold)	3D T1-w MRI	Binary Classification	AD *vs.* NC; AD *vs.* MCI; NC *vs.* MCI	Supervised Convolutional Neural Network (CNN)	AD *vs.* NC: 90.15%; AD *vs.* MCI: 87.30%; NC *vs.* MCI: 83.90%	AD *vs.* NC: 87.12%; AD *vs.* MCI: 85.30%; NC *vs.* MCI: 75.63%	AD *vs.* NC: 96.50%; AD *vs.* MCI: 90.20%; NC *vs.* MCI: 88.90%	-	-	-	Not mentioned
Sorour *et al*. [33]	Kaggle (Mild-Demented: 896, Moderate-Demented: 64, Very-Mild-Demented: 2240, Non-Demented: 3200)	Data Augmentation	80% training and 20% testing	MRI	Binary Classification	Demented (Mild, Moderate and Very-Mild-Demented) *vs.* Non-Demented	CNNs combined with LSTM	99.92%	100.00%	99.00%	100.00%	100.00%	-	Not mentioned
Tajammal *et al*. [34]	ADNI (AD: 1566, CN: 1376, EMCI: 1471, MCI: 1260, LMCI: 856, SMC: 1183)	Data augmentation	80% training, 20% testing	fMRI	Binary and Multi-class Classification	AD *vs.* CN; MCI *vs.* AD; CN *vs.* MCI; AD *vs.* SMC; EMCI *vs.* AD; LMCI *vs.* AD; CN *vs.* SMC; EMCI *vs.* CN; LMCI *vs.* CN; CN *vs.* EMCI *vs.* MCI *vs.* LMCI *vs.* SMC *vs.* AD	VGG-16, ResNet-18, AlexNet, Inception v1, and Custom CNN	AD *vs.* CN: 99.6%; MCI *vs.* AD: 99.4%; CN *vs.* MCI: 99.8%; AD *vs.* SMC:93.4%; EMCI *vs.* AD: 93.5%; LMCI *vs.* AD:91.3%; CN *vs.* SMC: 92.4%; EMCI *vs.* CN: 93.2%; LMCI *vs.* CN: 92.5%; CN *vs.* EMCI *vs.* MCI *vs.* LMCI *vs.* SMC *vs.* AD: 98.8%	----	AD *vs.* CN: 95.8%; MCI *vs.* AD: 96.6%; CN *vs.* MCI: 95.7%; AD *vs.* SMC:92.6%; EMCI *vs.* AD: 94.5%; LMCI *vs.* AD:90.2%; CN *vs.* SMC: 93.4%; EMCI *vs.* CN: 89.7%; LMCI *vs.* CN: 89.6%	---	AD *vs.* CN: 94.3%; MCI *vs.* AD: 96.2%; CN *vs.* MCI: 94.5%; AD *vs.* SMC:90.6%; EMCI *vs.* AD: 91.4%; LMCI *vs.* AD:89.7%; CN *vs.* SMC: 90.5%; EMCI *vs.* CN: 92.3%; LMCI *vs.* CN: 90.5%	-	Not mentioned
Ghaffari,Tavakoli, and Pirzad Jahromi [35]	ADNI (AD: 94, pMCI: 65, sMCI: 61, NC: 85), OASIS (AD: 15, NC: 15) and AIBL (AD:15, pMCI: 15, sMCI: 15, NC: 15)	Data augmentation	80% training, 10% validation, 10% testing	3D t1-w s-MRI	Binary and Multi-class Classification	NC *vs.* AD + pMCI + sMCI; NC *vs.* pMCI *vs.* sMCI *vs.* AD	Pre-trained CNN models with Trans- fer Learning (TL): ResNet101, Xcep- tion, InceptionV3	NC *vs.* AD + pMCI + sMC: 93.75% (ADNI), 93.33% (OASIS), 93.33% (AIBL); NC *vs.* pMCI *vs.* sMCI *vs.* AD: 93.75% (ADNI), 90.0% (AIBL)	-	-	-	-	NC *vs.* AD + pMCI + sMC: 92.0% (ADNI), 93.00% (OASIS), 95% (AIBL); NC *vs.* pMCI *vs.* sMCI *vs.* AD: 96.00% (ADNI), 93.00% (AIBL)	Not mentioned
Chabib, Had- jileontiadis and Shehhi [36]	Kaggle (ND: 3200, VMD: 2240, MID: 896, MOD: 64)	Leave-One-Group-Out Cross-Validation (LOGOCV) and k-fold cross-validation (10-fold and 5-fold)	80% training, 20% testing	MRI	Binary and Multi-class Classification	Non- Demented (ND) *vs.* Very Mild Demented (VMD); ND *vs.* VMD *vs.* MID *vs.* MOD	Deep Convolutional Curvelet Transform-based CNN (Deep- CurvMRI)	ND *vs.* VMD: 98.71%; ND *vs.* VMD *vs.* MID *vs.* MOD: 98.62%	ND *vs.* VMD: 98.50%; ND *vs.* VMD *vs.* MID *vs.* MOD: 98.50%	ND *vs.* VMD: 98.84%; ND *vs.* VMD *vs.* MID *vs.* MOD: 99.05%	ND *vs.* VMD: 99.25%; ND *vs.* VMD *vs.* MID *vs.* MOD: 99.21%	-	-	Curvelet Transform
Al-Otaibi *et al*. [37]	Kaggle (retreived from ADNI. For multi-class classification: CN: 1440, MCI: 2590, AD: 1124. for binary classification: AD: 965, MCI: 689)	ADASYN (Adaptive Synthetic Sampling)	80% training, 20% testing and 10-fold cross-validation	MRI	Binary and Multi-class Classification	AD *vs.* MCI; AD *vs.* MCI *vs.* CN	Dual Attention Convolutional AutoEncoder (DACNA)	AD *vs.* MCI: 99.22%; AD *vs.* MCI *vs.* CN: 98.30%	AD *vs.* MCI: 99.27%; AD *vs.* MCI *vs.* CN: 99.18%	AD *vs.* MCI: 99.27%; AD *vs.* MCI *vs.* CN: 98.32%	AD *vs.* MCI: 99.23%; AD *vs.* MCI *vs.* CN: 98.20%	AD *vs.* MCI: 99.28%; AD *vs.* MCI *vs.* CN: 98.18%	AD *vs.* MCI: 99.19%; AD *vs.* MCI *vs.* CN: 99.49%	Not mentioned
Thangavel, Natarajan and Preethaa [38]	Kaggle (CN: 3200, Very Mild Dementia: 2240, Mild Dementia: 896, Moderate Dementia: 87))	Data augmentation (Keras Image Data Generator) and 10-fold cross-validation	80% training, 20% testing	MRI	Multi-class Classification	Non-demented *vs.* very mild demented *vs.* mild demented *vs.* moderate demented	CNN-ResNet architecture with Modified Adam Optimization	98%	-	-	90%	---	-	Not mentioned
Boudi, He and Abd El Kader [39]	Kaggle (CN: 3200, Very Mild Dementia: 2240, Mild Dementia: 896, Moderate Dementia: 87))	Data augmentation (Keras Image Data Generator) and 10-fold cross-validation, SMOTE (Synthetic Minority Over-sampling Technique)	80% training, 10% validation, and 10% testing	MRI	Multi-class Classification	Non- Demented *vs.* Very Mild Demented *vs.* Mild Demented *vs.* Moderate Demented	Transfer Learning: ResNet50V2 (Best Model), VGG16, VGG19, DenseNet201	98.25%	-	98.00%	98.00%	98.00%	----	Grad-CAM
Pandey *et al*. [40]	ADNI (AD: 12028, MCI: 9604, CN: 13146) and OASIS (AD: 488, MCI: 6000, CN: 6000)	Data augmentation (Keras Image Data Generator)	80% training, 20% testing	3D T1-w MRI	Binary and Multi-class Classification	AD *vs.* CN; MCI *vs.* CN; AD *vs.*CN *vs.* MCI	Transfer learning: ResNet-50, ResNet-101 (Best Model), ResNet- 152, DenseNet-201, EfficientNet-B0	AD *vs.* CN: 92.34% (ADNI), 90.01% (OASIS); MCI *vs.* CN: 86.57% (ADNI), 86.87% (OASIS); AD *vs.* CN *vs.* MCI: 98.21% (ADNI), 97.45% (OASIS)	-	AD *vs.* CN: 90.02% (ADNI), 90.17% (OASIS); MCI *vs.* CN: 92.34% (ADNI), 88.76% (OASIS); AD *vs.* CN *vs.* MCI: 94.89% (ADNI), 93.67% (OASIS)	AD *vs.* CN: 92.89% (ADNI), 91.89% (OASIS); MCI *vs.* CN: 85.23% (ADNI), 81.23% (OASIS); AD *vs.* CN *vs.* MCI: 94.78% (ADNI), 93.45% (OASIS)	AD *vs.* CN: 90.12% (ADNI), 91.34% (OASIS); MCI *vs.* CN: 79.45% (ADNI), 78.99% (OASIS); AD *vs.* CN *vs.* MCI: 94.67% (ADNI), 93.12% (OASIS)	-	Grad-CAM
Parvatham and Maguluri [41]	Kaggle (2560 healthy subjects, very mild dementia: 1792, mild dementia: 717, moderate dementia: 52)	Data augmentation (15-fold-cross-validation)	80% training, 20% testing	T1 s-MRI	Binary Classification	Demented *vs.* Non- Demented	Hybrid CNN-SVM model	99.60%	99.40%	99.83%	99.58%	99.35%	-	Not mentioned
Basheera and Satya Sai Ram [42]	ADNI (Non-demented: 2560, very mild dementia: 1792, mild dementia: 717, moderate dementia: 52)	Only horizontal flipping	75% training, 25% testing and 10-fold cross-validation	T1 and T2 MRI	Binary and Multi-class Classification	AD *vs.* CN; AD *vs.* MCI; MCI *vs.* CN; AD *vs.* MCI *vs.* CN	Adaboost classifier using LM Filter Bank features	AD *vs.* CN: 84.24%; AD *vs.* MCI: 79.33%; AD *vs.* MCI *vs.* CN: 72.88%	AD *vs.* CN: 79.22%; AD *vs.* MCI: 80.00%; AD *vs.* MCI *vs.* CN: 58.88%	AD *vs.* CN: 89.85%; AD *vs.* MCI: 78.82%; AD *vs.* MCI *vs.* CN: 77.77%	-	-	-	Not mentioned
Fan *et al*. [43]	ADNI (AD: 108, LMCI: 163, EMCI: 261, NC: 213) and AIBL (AD: 13, NC: 13)	Oversampling	80% training, 20% testing, 5-fold cross-validation	3D T1 MRI	Binary and Multi-class Classification	AD *vs.* NC; NC *vs.* EMCI; EMCI *vs.* LMCI; LMCI *vs.* AD; NC *vs.* EMCI *vs.* LMCI *vs.* AD	U-net architecture with deep supervision and skip-connections	AD *vs.* NC: 95.71%; NC *vs.* EMCI: 87.98%; EMCI *vs.* LMCI: 90.14%; LMCI *vs.* AD: 90.05%; NC *vs.* EMCI *vs.* LMCI *vs.* AD: 86.47%	-	-	-	-	AD *vs.* NC: 0.89	Grad-CAM
Mahim *et al*. [44]	Kaggle (CN: 3200, Very Mild Dementia: 2240, Mild Dementia: 896, Moderate Dementia: 64) and ADNI (AD: 615, MCI: 1455, CN: 900)	Effective feature engineering	10-fold cross- validation and 80% training, 10% testing, and 10% validation	T1 MRI	Binary and Multi-class Classification	AD *vs.* CN; Demented *vs.* Healthy; No Dementia *vs.* Very Mild Dementia *vs.* Mild Dementia *vs.* Moderate Dementia	ViT-GRU hybrid model	Demented *vs.* Non-Demented: 99.69%; No Dementia *vs.* Very Mild Dementia *vs.* Mild Dementia *vs.* Moderate Dementia: 99.53%	Demented *vs.* Non-Demented: 99.47%; No Dementia *vs.* Very Mild Dementia *vs.* Mild Dementia *vs.* Moderate Dementia: 99.76%	Demented *vs.* Non-Demented: 99.53%; No Dementia *vs.* Very Mild Dementia *vs.* Mild Dementia *vs.* Moderate Dementia: 99.53%	Demented *vs.* Non-Demented: 99.53%; No Dementia *vs.* Very Mild Dementia *vs.* Mild Dementia *vs.* Moderate Dementia: 99.53%	Demented *vs.* Non-Demented: 99.53%; No Dementia *vs.* Very Mild De- mentia *vs.* Mild Dementia *vs.* Moderate De- mentia: 99.53%	-	LIME, SHAP ve Attention Map
Mehmood *et al*. [45].	ADNI (CN: 2520, MCI: 1995, LMCI: 3475, AD: 3475)	Data augmentation/feature extraction	80% training, 20% testing	2D T1 MRI	Binary classification	NC *vs.* AD; NC *vs.* LMCI; NC *vs.* MCI; MCI *vs.* AD; LMCI *vs.* AD	Siamese 4D- AlzNet model with transfer learning using Frozen VGG-16, Frozen VGG-19, and customized AlexNet	NC *vs.* AD: 95.07%; NC *vs.* LMCI: 96.75%; NC *vs.* MCI: 96.82%; MCI *vs.* AD: 95.43%; LMCI *vs.* AD: 79.16%	-	NC *vs.* AD: 92.51%; NC *vs.* LMCI: 95.93%; NC *vs.* MCI: 92.10%; MCI *vs.* AD: 94.85%; LMCI *vs.* AD: 76.36%	NC *vs.* AD: 95.90%; NC *vs.* LMCI: 97.22%; NC *vs.* MCI: 94.24%; MCI *vs.* AD: 96.45%; LMCI *vs.* AD: 86.05%	NC *vs.* AD: 99.56%; NC *vs.* LMCI: 98.56%; NC *vs.* MCI: 96.49%; MCI *vs.* AD: 98.12%; LMCI *vs.* AD: 98.56%	-	Not mentioned
Chatterjee and Byun [46]	OASIS (Subjects: 150)	Feature selection, imputation	70% training, 30% testing and 5-fold cross-validation	T1-w MRI	Binary Classification	Demented *vs.* Non- Demented	Voting Ensemble of base classifiers: SVM, KNN, Logistic Regression, Naive Bayes	96.43%	96.81%	94.64%	-	-	97.26%	Not mentioned
Aparna and Rao [47]	ADNI (All MRIs: 1296)	Data augmentation	95% training, 5% testing and 5-fold cross-validation	T1-w MRI	Multi-class Classification	CN *vs.* LMCI *vs.* EMCI *vs.* MCI *vs.* AD	Hybrid Xception and FractalNet deep learning architecture	99.06%	-	98.30%	-	99.72%	98.72%	Not mentioned
Cao *et al*. [48]	ADNI (Subject-based: NC: 172, EMCI: 188, LMCI: 161)	No need data is balanced	10-fold cross-validation (90% train, 10% test for each fold)	rs-fMRI and BOLD (Blood-oxygenation-level–dependent imaging) signals	Binary and Multi-class Classification	NC *vs.* EMCI; EMCI *vs.* LMCI; NC *vs.* EMCI *vs.* LMCI	S4D (Diagonal-Structured State-Space Sequence Model) integrated into a deep learning framework	NC *vs.* EMCI: 87.4%; EMCI *vs.* LMCI: 85.0%; NC *vs.* EMCI *vs.* LMCI: 77.9%	-	NC *vs.* EMCI: 86.4%; EMCI *vs.* LMCI: 89.0%; NC *vs.* EMCI *vs.* LMCI: 80.1%	-	-	NC *vs.* EMCI: 0.95; EMCI *vs.* LMCI: 0.93; NC *vs.* EMCI *vs.* LMCI: 0.92	Pointwise Convolutional

## Data Availability

All the data and supporting information are provided within the article.

## Footnotes

1
https://www.alz.org/alzheimers-dementia/what-is-alzheimers

## LIST OF ABBREVIATIONS

AD: Alzheimer’s Disease
ADASYN: Adaptive Synthetic Sampling
ADNI: Alzheimer’s Disease Neuroimaging Initiative
aMCI: Amnestic Mild Cognitive Impairment
AUC: Area Under the Curve
CAD: Computer-Aided Diagnosis
cAD: Converter Alzheimer’s Disease
CN: Cognitively Normal
CSF: Cerebrospinal Fluid
CNN: Convolutional Neural Network
DCNN: Deep Convolutional Neural Network
FAQ: Functional Activities Questionnaire
fMRI: Functional Magnetic Resonance Imaging
FL: Focal Loss
GANs: Generative Adversarial Networks
GDL: Generalized Dice Loss
LOGOCV: Leave-One-Group-Out Cross-Validation
MCI: Mild Cognitive Impairment
mD: Moderate Dementia
miD: Mild Dementia
MMSE: Mini-Mental State Examination
NC: Normal Cognition
OASIS: Open Access Series of Imaging Studies
oMCI: Other Mild Cognitive Impairment
PET: Positron Emission Tomography
pMCI: Progressive Mild Cognitive Impairment
PRISMA: Preferred Reporting Items for Systematic Reviews and Meta-Analyses
RF: Random Forest
ROC: Receiver Operating Characteristic
SES: Socioeconomic Status
sMCI: Stable Mild Cognitive Impairment
SMOTE: Synthetic Minority Over-sampling Technique
STARD: Standards for Reporting Diagnostic Accuracy Studies
SVM: Support Vector Machine
vmiD: Very Mild Dementia

## STANDARDS OF REPORTING

PRISMA guidelines were followed.

## References

[1] Morris J.C., Storandt M., Miller J.P., McKeel D.W., Price J.L., Rubin E.H., Berg L. (2001). Mild cognitive impairment represents early-stage Alzheimer disease.. Arch. Neurol..

[2] Petersen R. (2009). Early diagnosis of Alzheimer’s disease: Is MCI too late?. Curr. Alzheimer Res..

[3] Thung K.H., Wee C.Y., Yap P.T., Shen D. (2016). Identification of progressive mild cognitive impairment patients using incomplete longitudinal MRI scans.. Brain Struct. Funct..

[4] Goenka N., Tiwari S. (2021). Deep learning for Alzheimer prediction using brain biomarkers.. Artif. Intell. Rev..

[5] Henriques A.D., Benedet A.L., Camargos E.F., Rosa-Neto P., Nóbrega O.T. (2018). Fluid and imaging biomarkers for Alzheimer’s disease: Where we stand and where to head to.. Exp. Gerontol..

[6] Vos T., Allen C., Arora M., Barber R.M., Bhutta Z.A., Brown A., Carter A., Casey D.C., Charlson F.J., Chen A.Z., Coggeshall M., Cornaby L., Dandona L., Dicker D.J., Dilegge T., Erskine H.E., Ferrari A.J., Fitzmaurice C., Fleming T., Forouzanfar M.H., Fullman N., Gething P.W., Goldberg E.M., Graetz N., Haagsma J.A., Hay S.I., Johnson C.O., Kassebaum N.J., Kawashima T., Kemmer L., Khalil I.A., Kinfu Y., Kyu H.H., Leung J., Liang X., Lim S.S., Lopez A.D., Lozano R., Marczak L., Mensah G.A., Mokdad A.H., Naghavi M., Nguyen G., Nsoesie E., Olsen H., Pigott D.M., Pinho C., Rankin Z., Reinig N., Salomon J.A., Sandar L., Smith A., Stanaway J., Steiner C., Teeple S., Thomas B.A., Troeger C., Wagner J.A., Wang H., Wanga V., Whiteford H.A., Zoeckler L., Abajobir A.A., Abate K.H., Abbafati C., Abbas K.M., Abd-Allah F., Abraham B., Abubakar I., Abu-Raddad L.J., Abu-Rmeileh N.M.E., Ackerman I.N., Adebiyi A.O., Ademi Z., Adou A.K., Afanvi K.A., Agardh E.E., Agarwal A., Kiadaliri A.A., Ahmadieh H., Ajala O.N., Akinyemi R.O., Akseer N., Al-Aly Z., Alam K., Alam N.K.M., Aldhahri S.F., Alegretti M.A., Alemu Z.A., Alexander L.T., Alhabib S., Ali R., Alkerwi A., Alla F., Allebeck P., Al-Raddadi R., Alsharif U., Altirkawi K.A., Alvis-Guzman N., Amare A.T., Amberbir A., Amini H., Ammar W., Amrock S.M., Andersen H.H., Anderson G.M., Anderson B.O., Antonio C.A.T., Aregay A.F., Ärnlöv J., Artaman A., Asayesh H., Assadi R., Atique S., Avokpaho E.F.G.A., Awasthi A., Quintanilla B.P.A., Azzopardi P., Bacha U., Badawi A., Balakrishnan K., Banerjee A., Barac A., Barker-Collo S.L., Bärnighausen T., Barregard L., Barrero L.H., Basu A., Bazargan-Hejazi S., Beghi E., Bell B., Bell M.L., Bennett D.A., Bensenor I.M., Benzian H., Berhane A., Bernabé E., Betsu B.D., Beyene A.S., Bhala N., Bhatt S., Biadgilign S., Bienhoff K., Bikbov B., Biryukov S., Bisanzio D., Bjertness E., Blore J., Borschmann R., Boufous S., Brainin M., Brazinova A., Breitborde N.J.K., Brown J., Buchbinder R., Buckle G.C., Butt Z.A., Calabria B., Campos-Nonato I.R., Campuzano J.C., Carabin H., Cárdenas R., Carpenter D.O., Carrero J.J., Castañeda-Orjuela C.A., Rivas J.C., Catalá-López F., Chang J-C., Chiang P.P-C., Chibueze C.E., Chisumpa V.H., Choi J-Y.J., Chowdhury R., Christensen H., Christopher D.J., Ciobanu L.G., Cirillo M., Coates M.M., Colquhoun S.M., Cooper C., Cortinovis M., Crump J.A., Damtew S.A., Dandona R., Daoud F., Dargan P.I., das Neves J., Davey G., Davis A.C., Leo D.D., Degenhardt L., Gobbo L.C.D., Dellavalle R.P., Deribe K., Deribew A., Derrett S., Jarlais D.C.D., Dharmaratne S.D., Dhillon P.K., Diaz-Torné C., Ding E.L., Driscoll T.R., Duan L., Dubey M., Duncan B.B., Ebrahimi H., Ellenbogen R.G., Elyazar I., Endres M., Endries A.Y., Ermakov S.P., Eshrati B., Estep K., Farid T.A., Farinha C.S.S., Faro A., Farvid M.S., Farzadfar F., Feigin V.L., Felson D.T., Fereshtehnejad S-M., Fernandes J.G., Fernandes J.C., Fischer F., Fitchett J.R.A., Foreman K., Fowkes F.G.R., Fox J., Franklin R.C., Friedman J., Frostad J., Fürst T., Futran N.D., Gabbe B., Ganguly P., Gankpé F.G., Gebre T., Gebrehiwot T.T., Gebremedhin A.T., Geleijnse J.M., Gessner B.D., Gibney K.B., Ginawi I.A.M., Giref A.Z., Giroud M., Gishu M.D., Giussani G., Glaser E., Godwin W.W., Gomez-Dantes H., Gona P., Goodridge A., Gopalani S.V., Gotay C.C., Goto A., Gouda H.N., Grainger R., Greaves F., Guillemin F., Guo Y., Gupta R., Gupta R., Gupta V., Gutiérrez R.A., Haile D., Hailu A.D., Hailu G.B., Halasa Y.A., Hamadeh R.R., Hamidi S., Hammami M., Hancock J., Handal A.J., Hankey G.J., Hao Y., Harb H.L., Harikrishnan S., Haro J.M., Havmoeller R., Hay R.J., Heredia-Pi I.B., Heydarpour P., Hoek H.W., Horino M., Horita N., Hosgood H.D., Hoy D.G., Htet A.S., Huang H., Huang J.J., Huynh C., Iannarone M., Iburg K.M., Innos K., Inoue M., Iyer V.J., Jacobsen K.H., Jahanmehr N., Jakovljevic M.B., Javanbakht M., Jayaraman S.P., Jayatilleke A.U., Jee S.H., Jeemon P., Jensen P.N., Jiang Y., Jibat T., Jimenez-Corona A., Jin Y., Jonas J.B., Kabir Z., Kalkonde Y., Kamal R., Kan H., Karch A., Karema C.K., Karimkhani C., Kasaeian A., Kaul A., Kawakami N., Keiyoro P.N., Kemp A.H., Keren A., Kesavachandran C.N., Khader Y.S., Khan A.R., Khan E.A., Khang Y-H., Khera S., Khoja T.A.M., Khubchandani J., Kieling C., Kim P., Kim C., Kim D., Kim Y.J., Kissoon N., Knibbs L.D., Knudsen A.K., Kokubo Y., Kolte D., Kopec J.A., Kosen S., Kotsakis G.A., Koul P.A., Koyanagi A., Kravchenko M., Defo B.K., Bicer B.K., Kudom A.A., Kuipers E.J., Kumar G.A., Kutz M., Kwan G.F., Lal A., Lalloo R., Lallukka T., Lam H., Lam J.O., Langan S.M., Larsson A., Lavados P.M., Leasher J.L., Leigh J., Leung R., Levi M., Li Y., Li Y., Liang J., Liu S., Liu Y., Lloyd B.K., Lo W.D., Logroscino G., Looker K.J., Lotufo P.A., Lunevicius R., Lyons R.A., Mackay M.T., Magdy M., Razek A.E., Mahdavi M., Majdan M., Majeed A., Malekzadeh R., Marcenes W., Margolis D.J., Martinez-Raga J., Masiye F., Massano J., McGarvey S.T., McGrath J.J., McKee M., McMahon B.J., Meaney P.A., Mehari A., Mejia-Rodriguez F., Mekonnen A.B., Melaku Y.A., Memiah P., Memish Z.A., Mendoza W., Meretoja A., Meretoja T.J., Mhimbira F.A., Millear A., Miller T.R., Mills E.J., Mirarefin M., Mitchell P.B., Mock C.N., Mohammadi A., Mohammed S., Monasta L., Hernandez J.C.M., Montico M., Mooney M.D., Moradi-Lakeh M., Morawska L., Mueller U.O., Mullany E., Mumford J.E., Murdoch M.E., Nachega J.B., Nagel G., Naheed A., Naldi L., Nangia V., Newton J.N., Ng M., Ngalesoni F.N., Nguyen Q.L., Nisar M.I., Pete P.M.N., Nolla J.M., Norheim O.F., Norman R.E., Norrving B., Nunes B.P., Ogbo F.A., Oh I-H., Ohkubo T., Olivares P.R., Olusanya B.O., Olusanya J.O., Ortiz A., Osman M., Ota E., Pa M., Park E-K., Parsaeian M., de Azeredo Passos V.M., Caicedo A.J.P., Patten S.B., Patton G.C., Pereira D.M., Perez-Padilla R., Perico N., Pesudovs K., Petzold M., Phillips M.R., Piel F.B., Pillay J.D., Pishgar F., Plass D., Platts-Mills J.A., Polinder S., Pond C.D., Popova S., Poulton R.G., Pourmalek F., Prabhakaran D., Prasad N.M., Qorbani M., Rabiee R.H.S., Radfar A., Rafay A., Rahimi K., Rahimi-Movaghar V., Rahman M., Rahman M.H.U., Rahman S.U., Rai R.K., Rajsic S., Ram U., Rao P., Refaat A.H., Reitsma M.B., Remuzzi G., Resnikoff S., Reynolds A., Ribeiro A.L., Blancas M.J.R., Roba H.S., Rojas-Rueda D., Ronfani L., Roshandel G., Roth G.A., Rothenbacher D., Roy A., Sagar R., Sahathevan R., Sanabria J.R., Sanchez-Niño M.D., Santos I.S., Santos J.V., Sarmiento-Suarez R., Sartorius B., Satpathy M., Savic M., Sawhney M., Schaub M.P., Schmidt M.I., Schneider I.J.C., Schöttker B., Schwebel D.C., Scott J.G., Seedat S., Sepanlou S.G., Servan-Mori E.E., Shackelford K.A., Shaheen A., Shaikh M.A., Sharma R., Sharma U., Shen J., Shepard D.S., Sheth K.N., Shibuya K., Shin M-J., Shiri R., Shiue I., Shrime M.G., Sigfusdottir I.D., Silva D.A.S., Silveira D.G.A., Singh A., Singh J.A., Singh O.P., Singh P.K., Sivonda A., Skirbekk V., Skogen J.C., Sligar A., Sliwa K., Soljak M., Søreide K., Sorensen R.J.D., Soriano J.B., Sposato L.A., Sreeramareddy C.T., Stathopoulou V., Steel N., Stein D.J., Steiner T.J., Steinke S., Stovner L., Stroumpoulis K., Sunguya B.F., Sur P., Swaminathan S., Sykes B.L., Szoeke C.E.I., Tabarés-Seisdedos R., Takala J.S., Tandon N., Tanne D., Tavakkoli M., Taye B., Taylor H.R., Ao B.J.T., Tedla B.A., Terkawi A.S., Thomson A.J., Thorne-Lyman A.L., Thrift A.G., Thurston G.D., Tobe-Gai R., Tonelli M., Topor-Madry R., Topouzis F., Tran B.X., Truelsen T., Dimbuene Z.T., Tsilimbaris M., Tura A.K., Tuzcu E.M., Tyrovolas S., Ukwaja K.N., Undurraga E.A., Uneke C.J., Uthman O.A., van Gool C.H., Varakin Y.Y., Vasankari T., Venketasubramanian N., Verma R.K., Violante F.S., Vladimirov S.K., Vlassov V.V., Vollset S.E., Wagner G.R., Waller S.G., Wang L., Watkins D.A., Weichenthal S., Weiderpass E., Weintraub R.G., Werdecker A., Westerman R., White R.A., Williams H.C., Wiysonge C.S., Wolfe C.D.A., Won S., Woodbrook R., Wubshet M., Xavier D., Xu G., Yadav A.K., Yan L.L., Yano Y., Yaseri M., Ye P., Yebyo H.G., Yip P., Yonemoto N., Yoon S-J., Younis M.Z., Yu C., Zaidi Z., Zaki M.E.S., Zeeb H., Zhou M., Zodpey S., Zuhlke L.J., Murray C.J.L. (2016). Global, regional, and national incidence, prevalence, and years lived with disability for 310 diseases and injuries, 1990–2015: A systematic analysis for the Global Burden of Disease Study 2015.. Lancet.

[7] Hsu D. (2017). Primary and secondary prevention trials in Alzheimer disease: Looking back, moving forward.. Curr Alzheimer Res.

[8] Page M.J., McKenzie J.E., Bossuyt P.M., Boutron I., Hoffmann T.C., Mulrow C.D., Shamseer L., Tetzlaff J.M., Akl E.A., Brennan S.E., Chou R., Glanville J., Grimshaw J.M., Hróbjartsson A., Lalu M.M., Li T., Loder E.W., Mayo-Wilson E., McDonald S., McGuinness L.A., Stewart L.A., Thomas J., Tricco A.C., Welch V.A., Whiting P., Moher D. (2021). The PRISMA 2020 statement: An updated guideline for reporting systematic reviews.. PLoS Med..

[9] Bossuyt P.M., Reitsma J.B., Bruns D.E. (2015). STARD 2015: an updated list of essential items for reporting diagnostic accuracy studies.. BMJ.

[10] Agarwal D., Berbis M.A., Martín-Noguerol T., Luna A., Garcia S.C.P., de la Torre-Díez I. (2022). End-to-end deep learning architectures using 3d neuroimaging biomarkers for early alzheimer’s diagnosis.. Mathematics.

[11] Biswas R., Gini J R. (2023). Multi-class classification of Alzheimer’s disease detection from 3D MRI image using ML techniques and its performance analysis.. Multimedia Tools Appl..

[12] Qin Z., Liu Z., Guo Q., Zhu P. (2022). 3D convolutional neural networks with hybrid attention mechanism for early diagnosis of Alzheimer’s disease.. Biomed. Signal Process. Control.

[13] El-Sappagh S., Alonso J.M., Islam S.M.R., Sultan A.M., Kwak K.S. (2021). A multilayer multimodal detection and prediction model based on explainable artificial intelligence for Alzheimer’s disease.. Sci. Rep..

[14] Loddo A., Buttau S., Di Ruberto C. (2022). Deep learning based pipelines for Alzheimer’s disease diagnosis: A comparative study and a novel deep-ensemble method.. Comput. Biol. Med..

[15] Alhudhaif A., Polat K. (2023). Residual block fully connected DCNN with categorical generalized focal dice loss and its application to Alzheimer’s disease severity detection.. PeerJ Comput. Sci..

[16] Awarayi N.S., Twum F., Hayfron-Acquah J.B., Owusu-Agyemang K. (2024). A bilateral filtering-based image enhancement for Alzheimer disease classification using CNN.. PLoS One.

[17] AbdulAzeem Y., Bahgat W.M., Badawy M. (2021). A CNN based framework for classification of Alzheimer’s disease.. Neural Comput. Appl..

[18] Walaa N. (2023). A meta-heuristic multi- objective optimization method for alzheimer’s disease detection based on multi-modal data.. Mathematics.

[19] Goyal P., Rani R., Singh K. (2024). A multilayered framework for diagnosis and classification of Alzheimer’s disease using transfer learned Alexnet and LSTM.. Neural Comput. Appl..

[20] Kaya M, Çetın-Kaya Y. (2024). A novel deep learning architecture optimization for multiclass classification of Alzheimer’s disease level.. IEEE Access.

[21] Islam F., Rahman M.H. (2023). A novel method for diagnosing alzheimer’s disease from mri scans using the resnet50 feature extractor and the svm classifier.. Int. J. Adv. Comput. Sci. Appl..

[22] Khan R., Qaisar Z.H., Mehmood A., Ali G., Alkhalifah T., Alturise F., Wang L. (2022). A practical multiclass classification network for the diagnosis of alzheimer’s disease.. Appl. Sci..

[23] El-Latif A.A.A., Chelloug S.A., Alabdulhafith M., Hammad M. (2023). Accurate detection of alzheimer’s disease using lightweight deep learning model on mri data.. Diagnostics.

[24] Pan D., Luo G., Zeng A., Zou C., Liang H., Wang J., Zhang T., Yang B. (2024). Adaptive 3dcnn-based interpretable ensemble model for early diagnosis of alzheimer’s disease.. IEEE Trans. Comput. Soc. Syst..

[25] Fareed MMS, Zikria S, Ahmed G ADD-Net: An effective deep learning model for early detection of Alzheimer disease in MRI scans.. IEEE Access.

[26] Khatri U., Kwon G.R. (2022). Alzheimer’s disease diagnosis and biomarker analysis using resting-state functional MRI functional brain network with multi-measures features and hippocampal subfield and amygdala volume of structural MRI.. Front. Aging Neurosci..

[27] Javed Mehedi Shamrat FM (2023). AlzheimerNet: An effective deep learning based proposition for Alzheimer’s disease stages classification from functional brain changes in magnetic resonance images.. IEEE Access.

[28] Salehi W., Baglat P., Gupta G., Khan S.B., Almusharraf A., Alqahtani A., Kumar A. (2023). An approach to binary classification of alzheimer’s disease using LSTM.. Bioengineering.

[29] Kumari R., Nigam A., Pushkar S. (2022). An efficient combination of quadruple biomarkers in binary classification using ensemble machine learning technique for early onset of Alzheimer disease.. Neural Comput. Appl..

[30] Goyal P., Rani R., Singh K. (2024). An efficient ranking-based ensembled multiclassifier for neurodegenerative diseases classification using deep learning.. J. Neural Transm..

[31] Gamal A, Elattar M, Selim S (2022). Automatic early diagnosis of Alzheimer’s disease using 3D deep ensemble approach.. IEEE Access.

[32] Turkson R.E., Qu H., Mawuli C.B., Eghan M.J. (2021). Eghan. Classification of alzheimer’s disease using deep convolutional spiking neural network.. Neural Process. Lett..

[33] Sorour S.E., El-Mageed A.A.A., Albarrak K.M., Alnaim A.K., Wafa A.A., El-Shafeiy E. (2024). Classification of Alzheimer’s disease using MRI data based on Deep Learning techniques.. J. King Saud Univ. Comput. Inf. Sci..

[34] Tajammal T., Khurshid S.K., Jaleel A., Qayyum Wahla S., Ziar R.A. (2023). Deep learning-based ensembling technique to classify alzheimer’s disease stages using functional MRI.. J. Healthc. Eng..

[35] Ghaffari H., Tavakoli H., Pirzad Jahromi G. (2022). Deep transfer learning–based fully automated detection and classification of Alzheimer’s disease on brain MRI.. Br. J. Radiol..

[36] Chabib CM, Hadjileontiadis LJ, Shehhi AA (2023). DeepCurvMRI: Deep convolutional curvelet transform-based MRI approach for early detection of Alzheimer’s disease.. IEEE Access.

[37] Al-Otaibi S, Mujahid M, Khan AR, Nobanee H, Alyami J, Saba T Dual attention convolutional autoencoder for diagnosis of Alzheimer’s disorder in patients using neuroimaging and MRI features.. IEEE Access.

[38] Thangavel P., Natarajan Y., Sri Preethaa K.R. (2023). EAD-DNN: Early Alzheimer’s disease prediction using deep neural networks.. Biomed. Signal Process. Control.

[39] Boudi A., He J., El Kader I.A. (2024). Enhancing alzheimer’s disease classification with transfer learning: Finetuning a pre-trained algorithm.. Curr. Med. Imaging.

[40] Pandey P.K., Pruthi J., Alzahrani S., Verma A., Zohra B. (2024). Enhancing healthcare recommendation: Transfer learning in deep convolutional neural networks for Alzheimer disease detection.. Front. Med..

[41] Parvatham N.K., Maguluri L.P. (2024). Improved decision support system for alzheimer’s diagnosis using a hybrid machine learning approach with structural mri brain scans.. Int. J. Adv. Comput. Sci. Appl..

[42] Basheera S., Satya Sai Ram M. (2024). Leung-malik features and adaboost perform classification of alzheimer’s disease stages.. J. Inst. Electron. Telecommun. Eng..

[43] Fan Z., Li J., Zhang L., Zhu G., Li P., Lu X., Shen P., Shah S.A.A., Bennamoun M., Hua T., Wei W. (2021). U-net based analysis of MRI for Alzheimer’s disease diagnosis.. Neural Comput. Appl..

[44] Mahim SM, Ali MS, Hasan MO (2024). Unlocking the potential of XAI for improved Alzheimer’s disease detection and classification using a ViT-GRU model.. IEEE Access.

[45] Mehmood A., Shahid F., Khan R., Ibrahim M.M., Zheng Z. (2024). Utilizing siamese 4d-alznet and transfer learning to identify stages of alzheimer’s disease.. Neuroscience.

[46] Chatterjee S., Byun Y.C. (2022). Voting ensemble approach for enhancing alzheimer’s disease classification.. Sensors.

[47] Aparna M., Srinivasa Rao B. (2023). Xception-fractalnet: Hybrid deep learning based multi-class classification of alzheimer’s disease.. Comput. Mater. Continua.

[48] Cao T., Liu X., Du Z., Zhou J., Zheng J., Xu L. (2024). A diagonal structured-state-space-sequence-model based deep learning framework for effective diagnosis of mild cognitive impairment.. IEEE Sens. J..

